# Breast cancer subtyping from plasma proteins

**DOI:** 10.1186/1755-8794-6-S1-S6

**Published:** 2013-01-23

**Authors:** Fan Zhang, Jake Y Chen

**Affiliations:** 1 Department of Academic and Institutional Resources and Technology, University of North Texas Health Science Center, Fort Worth 76107, USA; 2 School of Informatics, Indiana University, Indianapolis, IN 46202, USA; 3 Department of Computer and Information Science, School of Science, Purdue University, Indianapolis, IN 46202, USA; 4 Indiana Center for Systems Biology and Personalized Medicine, Indianapolis, IN 46202, USA

## Abstract

**Background:**

Early detection of breast cancer in blood is both appealing clinically and challenging technically due to the disease's illusive nature and heterogeneity. Today, even though major breast cancer subtypes have been characterized, i.e., luminal A, luminal B, HER2+, and basal-like, little is known about the heterogeneity of breast cancer in blood, which could help to discover minimally invasive protein biomarkers with which clinical researchers can detect, classify, and monitor different breast cancer subtypes.

**Results:**

In this study, we performed an integrative pathway-assisted clustering analysis of breast cancer subtypes from plasma proteome samples collected from 80 patients diagnosed with breast cancer and 80 healthy women. First, four breast cancer subtypes and additionally unknown subtype (according to existing annotation) were determined based on pathology lab test results in primary tumors of enrolled patients. Next, we developed and applied four distance metrics, i.e., Protein Intensity, Q-Value, Pathway Profile, and Distance Score Function, to measure and characterize these cancer subtypes. Then, we developed a permutation test to evaluate the significant protein level changes in each biological pathway for each breast cancer subtype, using q-value. Lastly, we developed a pathway-protein matrix for each of the four distance methods to estimate the distance between breast cancer subtypes, for which further Pathway Association Network analysis were performed.

**Conclusions:**

We found that 1) the luminal group (luminal A and luminal B) are clustered together, as well as the basal group (basal-like and HER2+) and 2) luminal A and luminal B are more close to each other than basal-like and HER2+ to each other. Our results were consistent with a recent independent breast cancer research from the Cancer Genome Atlas Network using genomic DNA copy number arrays, DNA methylation, exome sequencing, messenger RNA arrays, microRNA sequencing and reverse-phase protein arrays. Our results showed that changes of different breast cancer subtypes at the pathway level are more profound and less variable than those at the molecular level. Similar subtypes share distinct yet similar pathway activation networks, while dissimilar subtypes are different also at the level of pathway activation networks. The results also showed that distance or similarity of cancer subtypes based on pathway analysis might be able to provide further insight into the intrinsic relationship of breast cancer subtypes. We believe integrative pathway-assisted proteomics analysis described here can become a model for reliable clustering or classification of other cancer subtypes.

## Background

Early detection and early intervention are keys to successful treatment of breast cancer, the second most common type of cancer after lung cancer worldwide. The American Cancer Society estimated that, in the United States alone, there will be about 226,870 new cases of invasive breast cancer, about 63,300 new cases of carcinoma in situ (CIS), and about 39,510 breast cancer deaths for 2012.

Functional genomics studies using DNA Microarrays or tandem mass spectrometry have been shown effective in differentiating between breast cancer tissues and normal tissues, by measuring thousands of differentially expressed genes or proteins simultaneously [1-3]. However, early detection of breast cancer in blood are both appealing clinically and challenging technically, partly because of 1) lack of routine blood test to screen early-stage breast cancers and 2) the fact that breast cancer is not a single homogeneous disease but consists of multiple disease subtypes, each arising from a distinct molecular mechanism and having a distinct clinical progression path [4]. Accurate classification of breast cancer subtypes, coupled with early detection, is therefore critical to effective cancer treatment, because mechanistically homogeneous breast cancer subtypes are easier to be distinguished from non-cancer conditions or treated than the entire heterogeneous breast cancer group.

Breast cancer disease heterogeneity has been confirmed at the gene expression level and categorized into five molecular subtypes: luminal A, luminal B, HER2+, basal-like, and normal-like, each with distinct gene expression patterns and prognosis [5]. For example, Perou et al. [6] originally showed that ER status divided breast tumors in two different branches, each arising from one of the two types of breast cancer cells: basal (ER-negative) and luminal (ER-positive) cells. ER-positive tumors represent 34-66% of all breast cancers. Tumors in the ER-positive group have expression patterns reminiscent of the luminal epithelial cells of the breast. ER-negative breast cancers, which represent 30-45% of all breast cancer, are characterized by lack of Hormone Receptor expression and low to absent expression of some other luminal markers. Recent inclusion of a larger number of samples and meta-analysis showed that luminal tumors can be subdivided into luminal A and luminal B subtypes, and basal tumors into normal-like, basal, and HER2+[5,7].

Recent studies further established refined classification criteria among these subtypes, based on similarity measures of their gene expression profiles [7,8]. This new classification system has given researchers more significant insights into the pathogenesis and metastasis of tumors than conventional pathological classification methods can do. However, there are several limitations that restrict their clinical applications. First, the transcription of genes is the first stage of gene expression and doesn't actually represent the actual functional molecules in the cell. On the contrary, the proteome, the complete set of proteins produced by the genome at any one time, is much more complex and dynamic than either the genome or the transcriptome. And in proteomics the real functional molecules of the cell are being studied. Second, either the gene expression signatures or protein changes from different tumor samples can be highly variable [9]. Third, the overlap of these signatures or changes among different data sets has been poor. For example, a comparison of results from two breast cancer treatment prognostic studies that led to clinical adoption and commercialization, Mammaprint and OncoDX, [10,11] revealed a very limited overlap between them, with only 3 out of 70 or 76 genes in common. Lastly, recent studies showed that genes or proteins differentially expressed between the primary breast (PBT) and metastatic lymph node (MLN) can be quite different, further limiting the power of determining prognostic outcomes based on gene expression or protein change profiles of PBT alone [12,13].

To accurately classify breast cancer subtypes in the presence of inherent "Omics" data noises, new pathway-driven data analysis approaches combined with proteomics have become necessary. In the past, many statistical methods have been developed to improve identification of subsets of differentially expressed genes or proteins from transcriptome or proteome profiling experiments of breast cancer [14]. While statistical tests can point to genes or proteins significantly altered between different cancer states or subtypes, they do not readily explain the biological contexts of such changes. In contrast, it is becoming increasingly apparent that genes or proteins function through complex molecular interactions to each other [15-17]. For example, breast cancer cell growth can be driven by mutations that lead to the constituent activation of the oestrogen receptor pathway [18]. It has also been discovered that different breast cancer subtypes originate from separate pathways [19]. However, due to lack of human biological pathway databases with sufficient quality and data coverage, integrated pathway study of breast cancer subtypes, particularly for data derived from proteomics experiments, have not been previously reported.

In this study, we developed an integrative pathway-assisted proteomics analysis method to study biological pathway-level changes in human breast cancer related plasma proteins, which we characterized from liquid-chromatography coupled tandem mass spectrometry (LC-MS/MS) proteomics experiments of plasma samples collected from patients diagnosed with breast cancer and healthy individuals. We used the new Human Pathway Database (HPD) [20] and the Integrated Pathway Analysis Database for Systematic Enrichment Analysis (IPAD) [21] to gain information on nearly one thousand human biological pathways, pathway protein constituents, and pathway-pathway similarity relationships. We labeled breast cancer patients with one of the four subtypes (luminal A, luminal B, HER2+, and basal-like) and additionally unknown subtype, using currently established clinical classification criteria [7,8]. We studied the use of four measures to the classification of breast cancer plasma proteomic results: Protein Intensity, Q-Value, Pathway Profiling, and Distance Score Function. We developed a permutation test to evaluate significant protein level changes in each biological pathway for each breast cancer subtype, using Q-value. Lastly, we developed a pathway-protein matrix for each of the four distance methods to estimate the distance between breast cancer subtypes and further performed pathway association analysis for each subtype.

Our results showed that each breast cancer subtype may be associated with changes of many different plasma proteins; however, the changes of different cancer subtypes at the pathway level are more profound and less variable than those at the molecular level. Similar subtypes share distinct yet similar pathway activation networks, while dissimilar subtypes are different also at the level of pathway activation networks. The results also showed that distance or similarity of cancer subtypes based on pathway analysis might be able to provide further insight into the intrinsic relationship of breast cancer subtypes. We believe integrative pathway-assisted proteomics analysis described here can become a model for reliable classification of other cancer subtypes.

## Methods

### Materials

Ammonium carbonate, ammonium bicarbonate, urea, formic acid, lysozyme, 2-Iodoethanol, and triethylphosphine were all purchased from Sigma-Aldrich (St. Louis, MO, USA). Acetonitrile and MS grade water were purchased from Honey Burdick & Jackson (Morristown, NJ, USA). Trypsin was purchased from Worthington Biochemical Corporation (Lakewood, NJ, USA). Seppro tip IgY-12 and reagent kit were purchased from GenWay Biotech (San Diego, CA, USA).

### Human plasma samples

Two batches of plasma samples were collected by the Hoosier Oncology Group (HOG) (Indianapolis, IN, USA) (each contained 40 plasma samples from women with breast cancer and 40 plasma samples from healthy age-matched volunteer women as control). All patients involved in this study were diagnosed with a stage II or earlier breast cancer. Most patients had previously been treated with chemotherapy. All samples were collected with the same standard operating procedure and stored in a central repository in Indianapolis, IN, USA.

### Proteomics methods

Biomarker identification and characterization holds great promise for more precise diagnoses and for tailored therapies. The heterogeneity of human cancers and unmet medical needs in these diseases provides a compelling argument to focus biomarker development in cancer. Mass Spectrometry (MS)-based proteomics approaches have provided insight into biomarkers of cancer and other diseases with femtomole sensitivity and high analytical precision.

Label-free protein identification and protein quantitative analysis services were performed by professionals at the Protein Analysis and Research Center/Proteomics Core of Indiana University School of Medicine, co-located at Monarch Life Sciences, Inc, Indianapolis. For a thorough review of the principle and method developed and used, refer to the review by Wang *et al *[22].

Proteins were prepared and subjected to LC/MS/MS analysis. First, all samples were run on a Surveyor HPLC (ThermoFinnigan) with a C18 microbore column (Zorbax 300SBC18, 1 mm × 5 cm). Then, all tryptic peptides (100 μL or 20 μg) were injected onto the column in random order. Next, peptides were eluted with a linear gradient from 5% to 45% acetonitrile developed over 120 min at a flow rate of 50 μL/min, and the eluant was introduced into a ThermoFinnigan LTQ linear ion-trap mass spectrometer. Last, the data were collected in the "triple-play" mode (MS scan, Zoom scan, and MS/MS scan). The database searches was performed using both the X!Tandem and SEQUEST algorithms.

### Protein identification and quantification

The International Protein Index (IPI) was used to map and identify sequence IDs. A LC/MS-based label-free protein quantification software licensed from Eli Lilly and Company was used to perform the protein quantification. First, all extracted ion chromatograms (XIC) were aligned by retention time, after the raw files were acquired from the LTQ. Each aligned peak should match parent ion, charge state, daughter ions (MS/MS data) and retention time (within a one-minute window). If any of these parameters are not matched, the peak is disqualified from the quantification analysis. Then, after the alignment, the area-under-the-curve (AUC) from individually aligned peak was measured, normalized, and compared for their relative abundance using methods described in [14]. Last, we transformed all peak intensities to a log2 scale for quantile normalization. If multiple peptides have the same protein identification, their quantile normalized log2 intensities are averaged to obtain log2 protein intensities, which are fit by the analysis of variance (ANOVA) statistical model for each protein as *y_ijk_*:

(1) $$y_{ijk}=\mu +T_{j}+S_{k}+I_{i}+\varepsilon_{ijk},$$

where $I_{i}\sim N\left(0,\sigma_{1}^{2}\right),S_{k}\sim N\left(0,\sigma_{2}^{2}\right),\varepsilon_{jk}\sim N\left(0,\sigma^{2}\right)$, *μ *is the mean intensity value, *T_j _*is the fixed group effect (caused by the experimental conditions or treatments being evaluated), *S_k _*is the random sample effect (random effects from either individual biological samples or sample preparations), *I_i _*is the random replicate effect (random effects from replicate injections of the same sample), and *ε_ijk _*are the within-groups errors. All of the injections were in random order and the instrument was operated by the same operator. All random effects are assumed independent of each other and independent of the within-groups errors *ε_ijk_*.

### Statistics test

Statistical Significance was measured by a three-step method. First, we conducted a permutation test to calculate the permutation test p value (also called false discovery rate). Then we calculated the FDR adjusted p value. Last, we calculated the FDR q value using the Storey-Tibshirani method [23].

We chose three significance screening filters (cutoff1 *q*<0.2, cutoff2 *q*<0.1, and cutoff3 *q*<0.05) to select proteins where we estimated significant differences in the healthy samples and each of the cancer subtypes. The False Positive Rate (FPR) or expected proportion of false positive among the proteins with declared changes is FPR = qvalue × number of the proteins with declared changes.

### Permutation test

**Figure 1:**
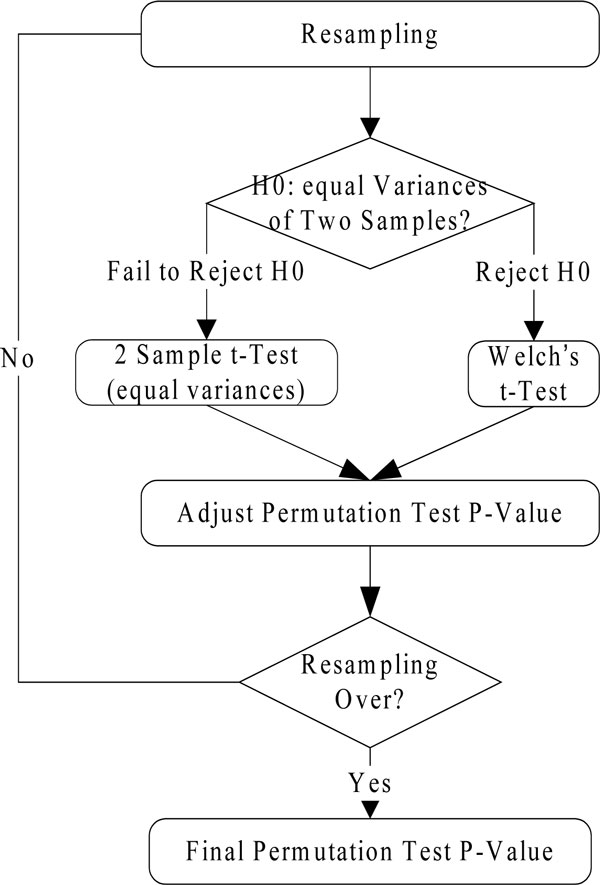
**flowchart of permutation test**.

We presented a permutation test which used two-sample t-statistics (for equal variance) and Welch's t-Test statistics (for unequal variance) to calculate the p-value of null hypothesis *H*_0_: *μ_h = _μ_c _*(h: healthy samples; c:cancer subtype) instead of using a single t-statistics (two-sample t-statistics or Welch's t-Test statistics) without considering sample variance in the traditional permutation test. Our test statistic is the difference between the mean of protein intensities in healthy samples and the mean of proteins intensities in cancer subtypes samples divided by the standard error of the mean. The statistical significance of protein with change was assessed by computing a permutation test p-value for each protein, representing the chance of observing a test statistic at least as large as the value actually obtained (Figure 1). All samples across the two groups for each protein were permuted 100,000 times and the complete set of t-tests between the two groups was performed for each permutation according to equations (2) and (3). The permutation p-value for a particular protein is the proportion of the permutations in which the permuted test p-value doesn't exceed the observed test p-value in absolute value.

After resampling, an F test is performed to compare the variances of two samples from normal populations. The F test's null hypothesis is that the variances of the two samples are equal. If the null hypothesis cannot be rejected, the 2 sample t-test with equal variance will be used. On the other hand, if the result of F-test indicates that the null hypothesis should be rejected, Welch's t-Test will be used.

For equal variance, 2 sample t-test statistics is calculated as

(2) $$t=\frac{\bar{X_{1}}-\bar{X_{2}}}{S_{X_{1}X_{2}}\cdot \sqrt{\frac{1}{n_{1}}+\frac{1}{n_{2}}}},$$

where $S_{X_{1}X_{2}}=\sqrt{\frac{\left(n_{1}-1\right)S_{X_{1}}^{2}+\left(n_{2}-1\right)S_{X_{2}}^{2}}{n_{1}+n_{2}-2}}\cdot S_{X_{1}X_{2}}$ is an estimator of the common standard deviation of the two samples. The degree of freedom for this test is *n*_1 _+ *n *_2 _-2.

For unequal variance, Welch's t-Test statistics is calculated as

(3) $$t=\frac{\bar{X_{1}}-\bar{X_{2}}}{S_{\bar{X_{1}}-\bar{X_{2}}}},$$

where $S_{\bar{X_{1}}-\bar{X_{2}}}=\sqrt{\frac{S_{1}^{2}}{n_{1}}+\frac{S_{2}^{2}}{n_{2}}}$ For use in significance testing, the distribution of the test statistic is approximated as being a Student's t distribution with the degrees of freedom calculated using

(4) $$df=\frac{{\left(s_{1}^{2}/n_{1}+s_{2}^{2}/n_{2}\right)}^{2}}{{\left(s_{1}^{2}/n_{1}\right)}^{2}/\left(n_{1}-1\right)+{\left(s_{2}^{2}/n_{2}\right)}^{2}/\left(n_{2}-1\right)}.$$

The permutation test p-value is adjusted at each resampling, if the permuted test p-value is less than or equal to the observed test p-value in absolute values, until the number of resampling reaches the number limitation of permutation.

### Cancer subtypes distances

#### Protein intensity

For protein intensity in a cancer subtype, a number of factors contribute to system variability: protein effects, subtype effects, individual effects and so on, plus interactions of these factors. Each of these main effects and interactions can be accounted for in a linear mixed model. Linear mixed model is primarily used to describe relationships between a response variable and some covariates in data that are grouped according to one or more classification factors. Linear mixed model estimates variance components using the residual maximum likelihood (REML) approach. We used R language and environment for statistical computing and graphics, which is a GNU project which is similar to the S language and environment (http://www.r-project.org), to fit linear mixed model.

A full factorial model was used to represent the fixed effect and two random effects which were used to account for individuals, subtypes and their interaction. The corresponding linear mixed model for the expression log ratios value *y_ijk _*for each gene/protein in the *j*th subtypes of the *i*th individual, is

(5) $$y_{ijk}=\mu +S_{j}+I_{i}+SI_{ij}+\varepsilon_{ijk},$$

Where $I_{i}\sim N\left(0,\sigma_{1}^{2}\right),\;SI_{ij}\sim N\left(0,\sigma_{2}^{2}\right),\;\varepsilon_{ijk}\sim N\left(0,\sigma^{2}\right)$. The fixed effects in Equation (5) are *μ*, the grand mean, and *S_j _*, the subtypes. The random effects in Equation (5) are *I_i_*, the individual random effect, and *SI_ij _*, the subtypes within individual random effect. All random effects were assumed independent of each other and independent of the within-groups errors *ε_ijk_*.

We defined the distance between subtype *s *and subtype *t *as

(6) $$d_{st}\text{ = 1}-Corr\text{(}S_{s}\text{,}S_{t}\text{),}$$

where *Corr *is the Pearson correlation coefficient.

#### Q-value

Based on the q value of protein change between healthy samples and cancer subtype samples, we defined the distance between subtype *s *and subtype *t *as

(7) $$d_{st}=1-Corr\left(qvalue_{s},qvalue_{t}\right),$$

where *Corr *is the Pearson correlation coefficient.

#### Pathway profiling

Based on the number of proteins in each pathway which is identified by Pathway Analysis, we defined the distance between subtype *s *and subtype *t *as

(8) $$d_{st}=1-Corr\left(N_{s},N_{t}\right),$$

where *Corr *is the Pearson correlation coefficient, *N_s _*is the vector of protein numbers in subtype *s*, and *N_t _*is the vector of protein numbers in subtype *t*.

#### Distance score function

We provided a measure of distance or similarity between cancer subtypes according to pathway-protein profiling. The dendrogram for each cancer subtype was then constructed based on the Hierarchical cluster analysis with the inferred cancer subtype distance matrix. The detailed process is described as follows.

Suppose a set of pathways in each cutoff, $O^{k}=\left\{o_{1},o_{2},\ldots ,o_{m^{k}}\right\}$, a set of proteins in each cutoff, $B^{k}=\left\{b_{1},b_{2},\ldots ,b_{n^{k}}\right\}$, where *k = *1,2,3 stands for three cutoffs (cutoff1 *q<*0.2, cutoff2 *q*<0.1, and cutoff3 *q*<0.05, respectively). Suppose a set of pathways in all three cutoffs, *O *= {*o*_1_, *o*_2_,..., *o_m_*}, and a set of proteins in all three cutoffs, *B *= {*b*_1_, *b*_2_,..., *b_n_*}, where *m *is the total number of pathways in all three cutoffs and *n *is the total number of proteins in all three cutoffs.

Pathway-protein matrices for the three cutoffs are $Q^{1}$, $Q^{2}$, and $Q^{3}$, respectively. In each Pathway-protein matrix, the row stands for a list of pathways, the column a list of proteins. Its *i, j *entry $q_{ij}^{k}\left(k=1,2,3;i=1,2,\ldots m;j=1,2,\ldots n\right)$ is *k *if the *j*th protein shows up in the *i*th pathway; otherwise 0.

The final pathway-protein matrix $Q={\left[q_{ij}\right]}_{m\times n}$ can be obtained by merging the three matrices $Q^{1}$, $Q^{2}$, and $Q^{3}$. Its element can be obtained by calculating the maximum value as shown in the following equation:

(9) $$q_{ij}=\underset{k=1,2,3}{\text{max}}q_{ij}^{k}.$$

Suppose the final pathway-protein matrix for the four subtypes (luminal A, luminal B, HER2+, and basal-like) and additionally unknown subtype is $Q^{l}$, *l *= 1,2,3,4,5 respectively. We define the distance between subtype *s *and subtype *t *as the average distance score for all pathway-protein pairs in $Q^{s}$ and $Q^{t}$, which is expressed as follows

(10) $$d_{st}=\frac{\underset{i,j}{\sum }score_{ij}\left(s,t\right)}{m\times n}=\frac{\underset{i,j}{\sum }f\left(q_{ij}^{s},q_{ij}^{t}\right)}{m\times n},$$

where *f *stands for the distance score function, $f\left(q_{ij}^{s},q_{ij}^{t}\right)=abs\left(q_{ij}^{s}-q_{ij}^{t}\right)$.

### Pathway analysis

Pathway Analysis are performed using the web tool HPD [24] and IPAD [21] we developed.

### Pathway association network

The pathway similarity measure is defined as the extent of overlaps, e.g., common number of genes/proteins, shared between two different pathways [25]. The pathway-pathway similarity score *S_i, j _*is defined as

(11) $$S_{i,j}=\frac{\left|P_{i}\cap P_{j}\right|}{\left|P_{i}\cup P_{j}\right|}i=1\ldots N,j=1\ldots N,$$

where, *N *denotes total number of pathways. *P_i _*and *P_j _*denote two different pathways, while |*P_i_*| and |*P_j_*| are the numbers of proteins in these two pathways. Their intersection *P_i _*∩ *P_j _*is the set of all proteins that appear in both *P_i _*and *P_j_*, while their union *P_i _*∪ *P_j _*is a set of all proteins either appearing in the *P_i _*or in the *P_j_*. Duplicates are eliminated in the intersection set and union set.

Cytoscape is used to visualize and associate pathway networks with pathway protein association matrix. The Edge line width is proportional to the similarity of the connected pathways. Node size is proportional to protein numbers in a pathway. Node color is proportional to the number of proteins with change between samples of each subtype and healthy samples in a pathway.

### Prediction performance of four distance metrics

In order to validate the pathway profiling, distance score function and permutation test we presented, we compare the true positives and accuracy of subtype prediction among Protein Intensity, Q-Value, Pathway Profiling with Traditional Permutation, Pathway Profiling, and Distance Score Function.

The nearest neighbor prediction is used to predict cancer subtypes with the 80 patients' data. The nearest neighbor prediction is a method that finds the closest (according to four different distance metrics) exemplar to the patient and predicts the subtype of the exemplar.

For protein intensity, we directly use the 80 patients' protein intensity data. Each subtype protein intensity is used as an exemplar. The distance between subtype *s *and patient *t *is defined as

(12) $$d_{st}\text{ = 1}-Corr\text{(}S_{s}\text{, }P_{t}\text{),}$$

where *Corr *is the Pearson correlation coefficient.

For Q-Value, we first compute the Q-Value for proteins in each patient by the statistical significance testing of the expression of the protein between all healthy women and the patient. Each subtype Q-Value is used as an exemplar. Pearson correlation coefficient distance measure is used to calculate how close each patient of the training set is to the five exemplars that are being examined.

The distance between subtype *s *and patient *t *is defined as

(13) $$d_{st}\text{ = 1}-Corr\text{(}qvalue_{s}\text{, }qvalue_{t}\text{),}$$

where *Corr *is the Pearson correlation coefficient.

For Pathway Profiling, based on the pathway-protein matrix for each patient and each subtype, we defined the distance between subtype *s *and patient *t *as

(14) $$d_{st}\text{ = 1}-Corr\text{(}N_{s}\text{,}N_{t}\text{),}$$

where *Corr *is the Pearson correlation coefficient, *N_s _*is the vector of protein numbers in subtype *s*, and *N_t _*is the vector of protein numbers in patient *t*. Each subtype's vector of protein numbers in pathway-protein matrix is used as an exemplar.

For Distance Score Function, the final pathway-protein matrix of each subtype is used as an exemplar. Similarly, we define the distance between subtype *s *and patient *t *as the average distance score for all pathway-protein pairs in $Q^{s}$ and $Q^{t}$, which is expressed as follows

(15) $$d_{st}=\frac{\underset{i,j}{\sum }score_{ij}\left(s,t\right)}{m\times n}=\frac{\underset{i,j}{\sum }f\left(q_{ij}^{s},q_{ij}^{t}\right)}{m\times n},$$

where *f *stands for the distance score function, $f\left(q_{ij}^{s},q_{ij}^{t}\right)=abs\left(q_{ij}^{s}-q_{ij}^{t}\right)$, *m *is the number of pathways, and *n *is the number of proteins.

The prediction process of Pathway Profiling with traditional Permutation is the same as that of Pathway Profiling except that the former uses traditional permutation and the latter uses the permutation test we presented.

## Results

### Plasma proteomics results for breast cancer cases and controls

The proteomics experiment included 160 plasma samples, 80 samples from women with breast cancer and 80 from healthy volunteer women which serve as controls. The plasma proteome set have 616 proteins which were mapped to 1458 UniprotID. Those proteins with no match in the Uniprot database were excluded from the pathway analysis.

### Histopathological data on clinical samples

Receptor status based on immunohistochemical (IHC) expression of the estrogen receptor (ER) or progesterone receptor (PR) and human epidermal growth factor receptor 2 (HER2) proteins was used to approximate subtype: ER+ or PR+ and HER2- (luminal A); ER- or PR- and HER2+ (luminal B); ER- and PR- and HER2+ (HER2+); and ER- and PR- and HER2- (basal-like). We have a total of 68 plasma samples from four different subtypes of breast cancer patients and the other 12 patient samples we called unknown subtype in which receptor statuses were missing. The number of patients, age, tumor, and grade, in each subtype, are shown in Table 1.

**Table 1:** clinical data summary for breast cancer patients

Subtype	Receptor Status	Number	Age	Cancer Type	Grade	Metastasis	Tumor Size
			1- <40	19 - INV	7 - G1	2 locally Recurrent	Min = 0.5
LuminalA	ER+ | PR+ & HER2-	24	20-[40,65]	4 - DCIS	10 - G2	3 Distant Metastasis	Max = 8
			3->65	1 - u	5 - G3	18 No	Mean = 2.550526
					2 - u		
			4- <40	9 - INV	2 - G1	1 locally Recurrent	Min = 0.2
LuminalB	ER+ | PR+ & HER2+	15	9-[40,65]	6 - DCIS	5 - G2	2 Distant Metastasis	max = 5.4
			2->65		7 - G3	12 No	mean = 2.205556
					1 - u		
			2- <40	8 - INV	0 - G1	0 locally Recurrent	min = 0.9
HER2Plus	ER- & PR- & HER2+	10	8-[40,65]	2 - DCIS	0 - G2	4 Distant Metastasis	max = 11
			0->65		10 -G3	6 No	mean = 3.8
			0- <40	15 - INV	1 - G1	3 locally Recurrent	min = 0.7
BasalLike	ER- & PR- & HER2-	19	18-[40,65]	4 - DCIS	3 - G2	3 Distant Metastasis	max = 6
			1->65		14 -G3	13 No	mean = 2.5
					1 - u		
			1- <40	2 - INV	1 - G1	0 locally Recurrent	
Unknown	Missing	12	9-[40,65]	2 - DCIS	1 - G2	2 Distant Metastasis	
			2->65	8 - u	0 - G3	1 No	
					10 - u		

INV: invasive; DCIS: Ductal Carcinoma in Situ; u: unknown

### Dendrograms of breast cancer subtypes

First we calculated the breast cancer subtypes distance based on Protein Intensity of 80 cancer samples. Each subtype's fixed effect was estimated by the Equation (5) and then the distance between two subtypes were obtained by the Equation (6).

Then, we calculated the breast cancer subtypes distance based on Q-Value. The Q-Value of protein change between healthy samples and cancer subtype samples were obtained by a three-step method mentioned in the statistics test section: 1) permutation test, 2) FDR adjusted p-value and 3) FDR Q-Value. The distance between two subtypes were obtained by the Equation (7).

Next, we calculated the breast cancer subtypes distance based on Pathway Profiling. Three cutoffs (cutoff1 *q*<0.2, cutoff2 *q*<0.1, and cutoff3 *q*<0.05) were chosen to identify proteins with significant changes in the healthy samples and each of the breast cancer subtypes. For example, the numbers of proteins with significant changes identified by cutoff1 were 780, 829, 818, 804, and 440, respectively, for luminal A, luminal B, HER2+, basal-like, and unknown.

The updated KEGG human pathway database consists of 215 pathways and 4955 gene IDs which we mapped to 22532 UniProt ID. We integrated the pathway database to our Oracle supported bio10G2 server and used the pathway web tool HPD [24] and IPAD [21] we developed to perform pathway analysis.

We got three pathway-protein matrices for each subtype, where the row is pathway and the column is protein biomarkers. Up-arrow stands for up-regulated, and down-arrow for down-regulated. The final pathway protein matrix after merging the three matrices is shown in Additional File 1. The distance between two subtypes were then obtained by the Equation (8).

Lastly, we calculated the breast cancer subtypes distance based on Distance Score Function. The Cancer Subtype Distance Matrix was obtained from Equation (10) (as shown in Table 2).

**Table 2:** cancer subtype distance matrix

Distance	LuminalA	LuminalB	HER2Plus	BasalLike	Unknown
LuminalA	0	151	573	589	1190
LuminalB	151	0	544	598	713
HER2Plus	573	544	0	302	990
BasalLike	589	598	302	0	1011
Unknown	1190	713	990	1011	0

**Figure 2:**
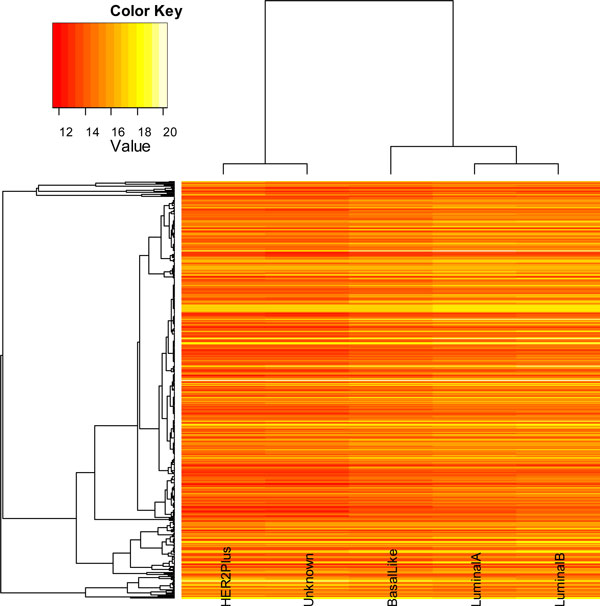
**heatmap of protein change across four subtypes (luminal a, luminal b, her2+, and basal-like) and additionally unknown subtype**. The y axis is protein markers. Each colored cell represents a protein intensity calculated by the Equation (5). The rows use hierarchical clustering with Euclidean distance. The columns use hierarchical clustering with protein intensity distance defined in the Equation (6).

**Figure 3:**
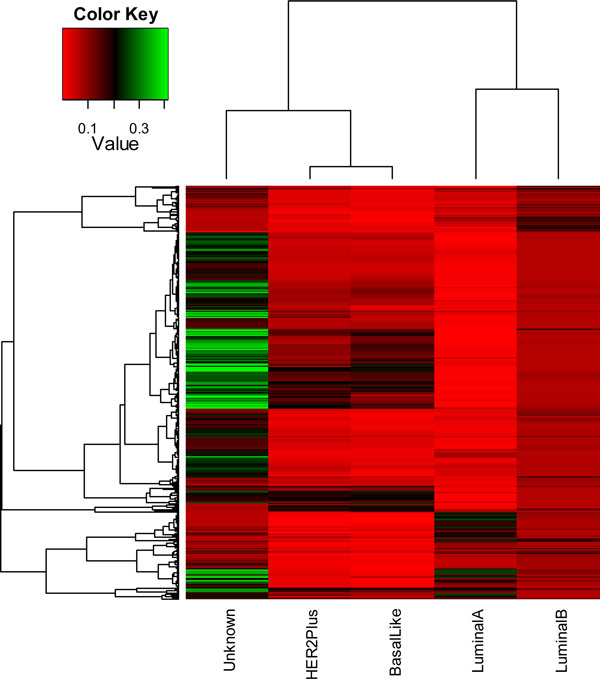
**heatmap of q value across four subtypes (luminal A, luminal B, HER2+, and basal-like) and additionally unknown subtype**. The y axis is protein markers. Each colored cell represents a Q value. The rows use hierarchical clustering with Euclidean distance. The columns use hierarchical clustering with Q-Value distance defined in the Equation (7).

**Figure 4:**
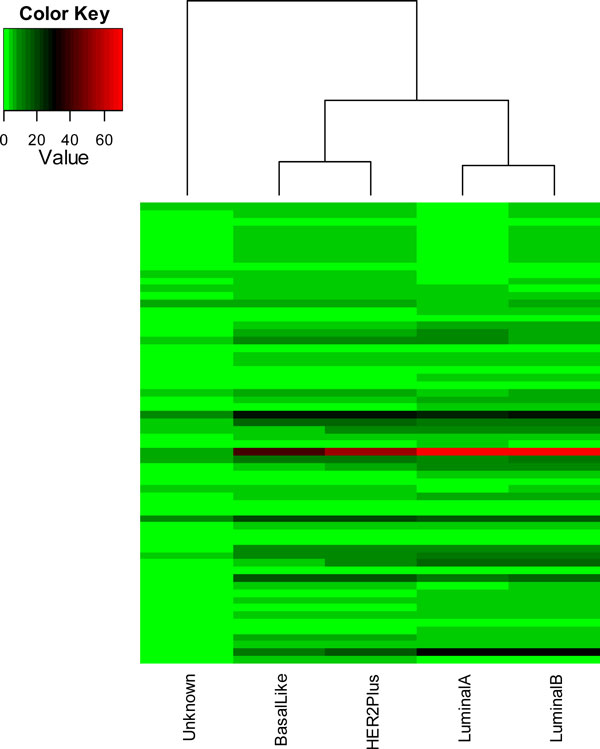
**pathway profiling across four subtypes (luminal A, luminal B, HER2+, and basal-like) and additionally unknown subtype(q-value < 0.1)**. The y axis is pathways. Each colored cell represents number of protein in a pathway. The rows use hierarchical clustering with Euclidean distance. The columns use hierarchical clustering with Pathway Profiling distance defined in the Equation (8).

**Figure 5:**
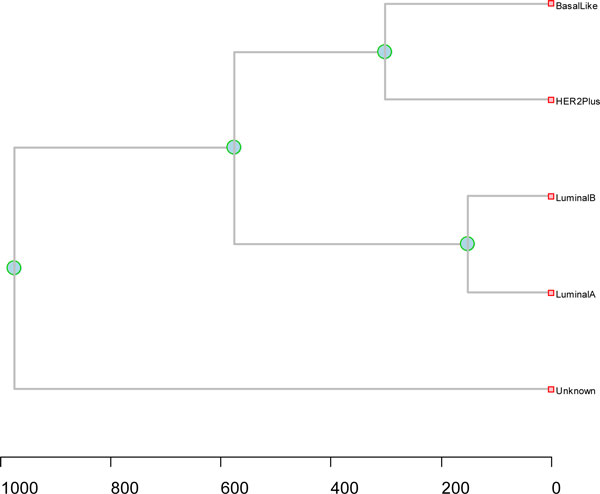
**hierarchical clustering of breast cancer subtypes**. The distances were computed by the Equation (10) according to the Distance Score Function.

**Figure 6:**
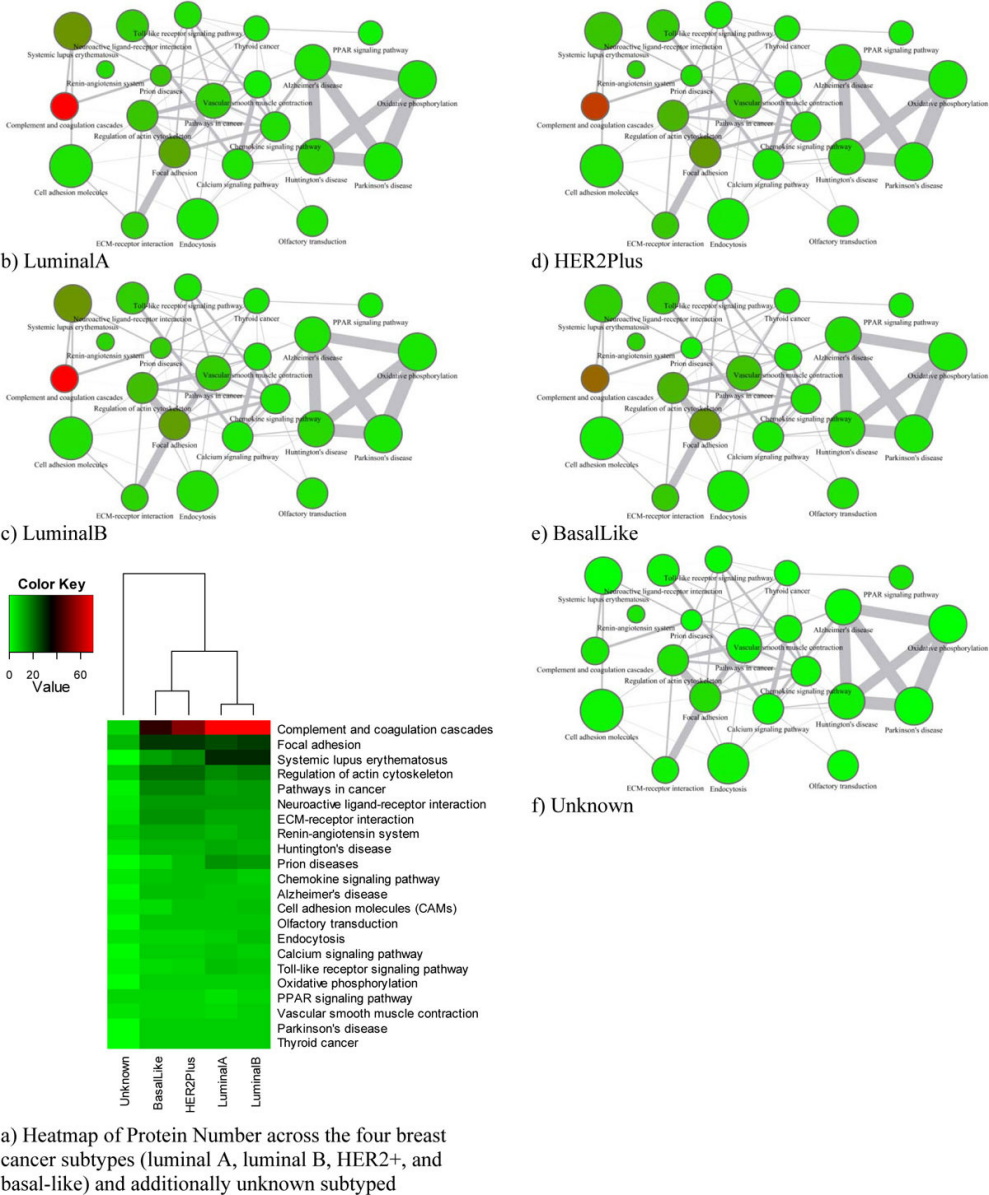
**pathway association network**. Edge line width is proportional to the connected pathways similarity. Node size is proportional to protein numbers in a pathway. Node color is proportional to the number of proteins with change between each subtype and control in a pathway.

We tested with the four distance measurement methods (Figures 2, 3, 4, 5). In the dendrogram of subtypes in Protein Intensity profiling (Figure 2), basal-like, luminal A and luminal B together are clustered together. The distance matrices based on Q-Value across four subtypes and additionally unknown subtype clearly distinguish between the luminal group (luminal A and luminal B) and the basal group (basal-like and HER2+) (Figure 3). Dendrograms in both Figure 4 and 5 show that 1) the luminal group (luminal A and luminal B) are clustered together, as well as the basal group (basal-like and HER2+), 2) the luminal group and the basal group are more close to each other than to unknown subtype, and 3) luminal A and luminal B are more close to each other than basal-like and HER2+ to each other(Figure 4, Figure 5 and Figure 6a).

### Evaluation

Our validations of the dendrogram at the pathway-level are three-folds. First we tested the prediction performance of four distance metrics. A distance measuring method with an accurate performance prediction can be used as a good clustering method. Then Literature curation was used to evaluate if our result is consistent with previous reports. Last, Pathway Association Network analysis was performed to mine the biological meaning of the pathway-assisted clustering, which in turn verified the feasibility of the method and the validity of the results.

**Figure 7:**
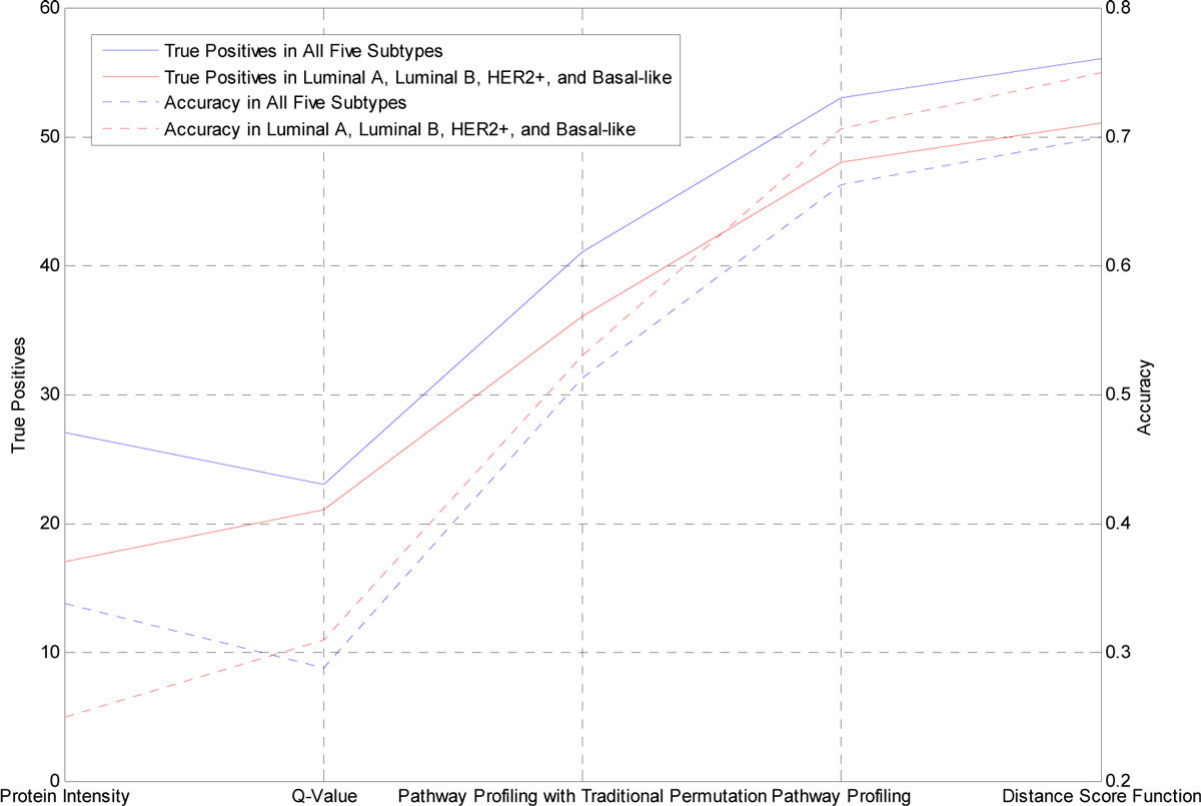
**true positives and accuracy with four metrics and traditional permutation**.

In our first evaluation, we validated the Pathway Profiling and Distance Score Function metrics method and permutation test we presented by comparing among the four distance metrics: Protein Intensity, Q-Value, Pathway Profiling, Distance Score Function, and Pathway Profiling with Traditional Permutation (Figure 7 and Additional File 2). The Distance measuring based on Pathway Profiling and Distance Score Function show fewer incorrect predictions and markedly improved accuracy (53 (66%) and 48 (71%) true positives for all five subtypes and all four known subtypes in Pathway Profiling; 56 (70%) and 51 (75%) true positives for all five subtypes and all four known subtypes in Distance Score Function). The pathway profiling distance measuring based on our permutation test shows improved accuracy compared to the pathway profiling distance measuring based on traditional permutation test (41 (51%) and 36 (53%) true positives for all five subtypes and all four known subtypes in Distance Score Function; 53 (66%) and 48 (71%) true positives for all five subtypes and all four known subtypes in Pathway Profiling).

**Figure 8:**
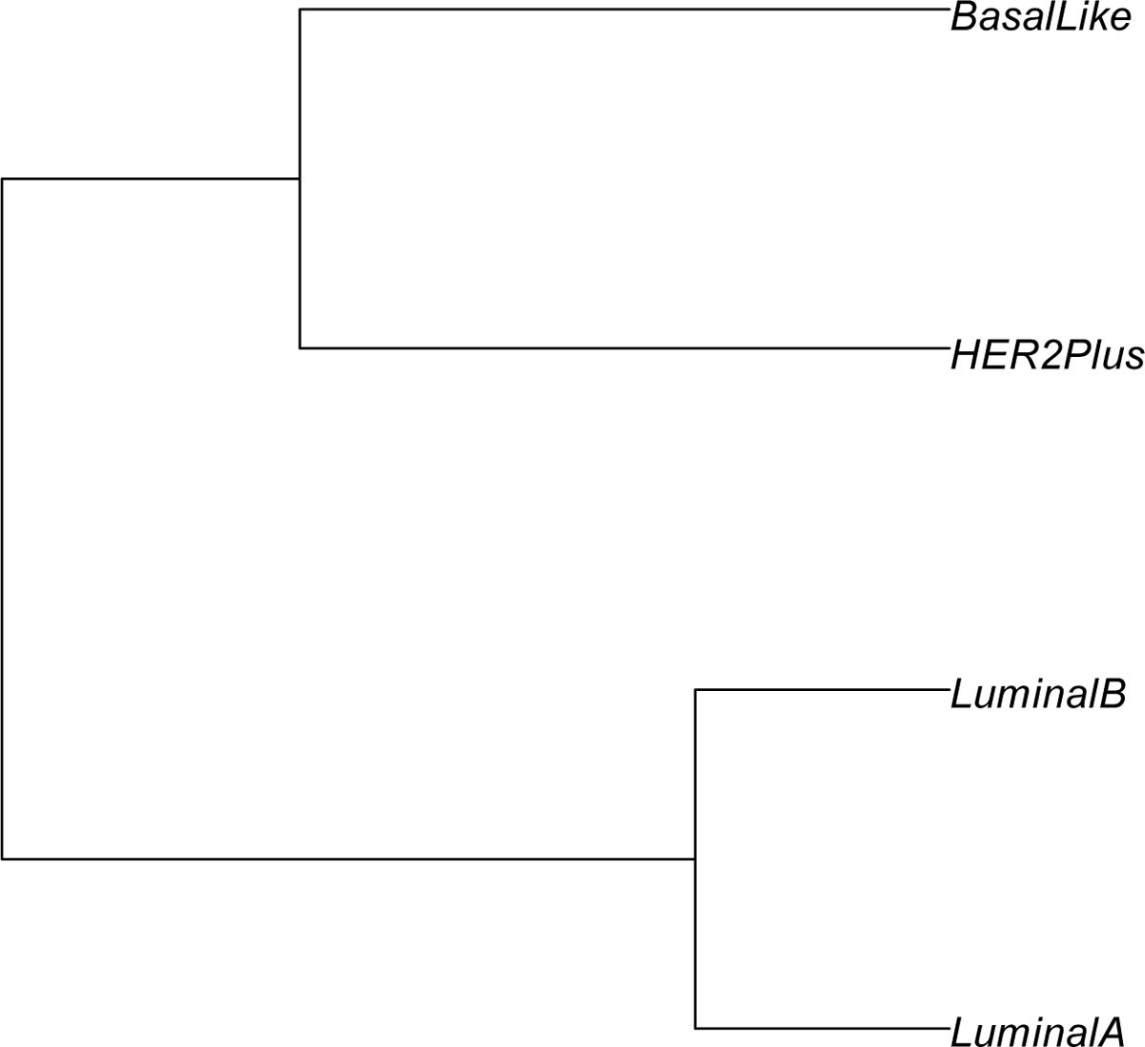
**dendrogram of primary breast cancers from the cancer genome atlas network**. The summary of the genomic, clinical and proteomic features of subtypes from the Cancer Genome Atlas Network, which includes the percentage of ER+/HER2-, HER2+, TNBCs, TP53 pathway, PIK3CA/PTEN pathway, RB1 pathway and DNA mutations, was used to draw the phylogenetic tree with hierarchical cluster analysis under Euclidean distance metric.

In the literature curation, we found our results (Figure 4, Figure 5, and Figure 6a) are consistent with previous findings. For example, the Cancer Genome Atlas Network analyzed primary breast cancers by genomic DNA copy number arrays, DNA methylation, exome sequencing, messenger RNA arrays, microRNA sequencing and reverse-phase protein arrays [26]. Their results from the genomic, clinical and proteomic features of breast cancer subtypes have established a high reliability compared to previous clustering results based singly on gene expression values. Based on the summary of the genomic, clinical and proteomic features of subtypes, which include the percentage of ER+/HER2-, HER2+, TNBCs, TP53 pathway, PIK3CA/PTEN pathway, RB1 pathway and DNA mutations, we performed hierarchical cluster analysis using the Euclidean distance metric (Figure 8). In the Figure 8, the luminal group (luminal A and luminal B) are grouped together and the basal group (basal-like and HER2+) are grouped together too, and luminal A and luminal B are more close to each other than basal-like and HER2+ to each other, which are consistent with our results in Figure 4, Figure 5 and Figure 6a.

Our results are also in accordance with some clustering analysis based on gene expression [27-29]. But clustering based on gene expression had diverse results. For example, Sørlie et al found that the luminal group and basal group were separated in 78 breast cancer tissue samples [27] and that Luminal B was categorized into basal group in 115 malignant breast tumors [30]. Marcotte et al. identified that HER2+ was more close to Luminal group than to basal-like [31]. The low reproducibility of the subtypes' distance measuring from the expression of individual genes in microarray experiments has led to the suggestion that experiments be analyzed in mass spectrometry and in terms of gene functions and pathways, in order to enhance the robustness of the results.

Lastly, we performed the Pathway Association Network Analysis to understand the intrinsic relationship between the subtypes of breast cancers which in turn validated our results of dendrogram based on Pathway Profiling and Distance Score Function.

The top 22 pathways were selected from the pathway protein matrices in the Additional File 1 with average protein number >5 (Figure 6a). In the Figure 4, pathway profiling with all pathways shows luminal A and luminal B hold together just a little bit tighter than basal-like and HER2+. After we filtered out those pathways with average protein number >5, we obtained a similar dendrogram (Figure 6a) to the dendrogram (Figure 5) we obtained using Distance Score Function, where luminal A and luminal B are more close to each other than basal-like and HER2+ to each other. This might suggest that functionally important pathways contribute more to the intrinsic relationship between the subtypes of breast cancer.

We then used the top 22 pathways to build a Pathway Association Network for each subtype as shown in Figures 6b-6f. The increasing red depth of nodes across the four subtypes (luminal A, luminal B, HER2+, and basal-like) and the unknown typing indicates the activated pathways due to tumor progression. The diagram of the Pathway Association Network provides visual cues about how the network changes with development of subtype. It shows that the changes of different breast cancer subtypes at the pathway level are more relatively conserved although each breast cancer subtype may be associated with change of many different proteins (Additional File 1). For example, 1) 15 out of the 22 pathways are in common across luminal A, luminal B, basal-like, and HER2+ (neuroactive ligand-receptor interaction, reninangiotensin system, huntington's disease, chemokine signaling pathway, alzheimer's disease, cell adhesion molecules, olfactory transduction, endocytosis, calcium signaling pathway, toll-like receptor signaling pathway, oxidative phosphorylation, ppar signaling pathway, vascular smooth muscle contraction, parkinson's disease, thyroid cancer); 2) an immune system pathway (complement and coagulation cascades), an immune disorders pathway (systemic lupus erythematosus), and a neurodegenerative diseases pathway (prion diseases) are more activated from the basal group to the luminal group, but the change of activated pathways within either the basal group or the luminal group is relatively conserved if compared with those change between the two groups. Luminal A and luminal B share similar network structure although focal adhesion pathway and regulation of actin cytoskeleton pathway can distinguish luminal A from luminal B. And basal-like and HER2+ share similar network structure, although complement and coagulation cascades pathway and systemic lupus erythematosus can distinguish basal-like and HER2+. The relationship of breast cancer subtypes shown in (Figures 6b-6f) are in consistent with the intrinsic relationships in the dendrograms we drew based on Pathway Profiling and Distance Score Function.

## Discussion

### Distances between breast cancer subtypes

Dendrogram linking gene/protein or subtype samples can be generated to show degrees of similarity between breast cancer subtypes. The dendrogram of subtypes in Protein Intensity profiling (Figure 2) grouped basal-like, luminal A and luminal B together. The distance matrix based on Q value across the four known subtypes produced similar hierarchical clustering with previously reported works from degree of similarity of gene expression profile between subtypes[5]. Basal-like should not be classified as Luminal tumor, because most of basal-like and HER2+ are significantly more likely to be grade III than the luminal A tumors [5,7,8]. Compared to the luminal A which appears to be associated with the best prognosis, the HER2 and basal-like have poor prognostic feature as defined by routine pathology methods [5,7,30,32]. The possible reason is that the degree of similarity between subtypes based on gene expression value or protein intensity might not correctly indicate the relationship between subtypes, (they may indicate well the relationship between genes or proteins). Although useful relationships between co-expressed genes or similar protein change patterns may be discovered from the variance of gene expression or protein intensity, genes with similar expression patterns or proteins with similar intensity change don't always mean they have similar functions or play roles in similar pathways; therefore, to some extent, they will miscategorize samples (subtypes).

Pathway Profiling and Distance Score Function both outperform the traditional gene expression or protein change profiling in measuring the distance or similarity of subtypes. Dendrograms in Figures 4 and 5 indicate: 1) the luminal group (luminal A and luminal B) are grouped together, as well as the basal group (basal-like and HER2+), 2) the luminal group and the basal group are more close to each other than to unknown subtype, and 3) luminal A and luminal B are more close to each other than basal-like and HER2+ to each other(Figure 4, Figure 5 and Figure 6a). As mentioned in evaluation section, these are consistent with the previous findings [26-29].

We believe the dendrograms we drew in Figures 5 and 6a relatively accurately reflects the intrinsic relationship between the four breast cancer subtypes. For example, the survival analysis of breast cancer subtypes found that the breast cancer subtypes also differed significantly in breast cancer specific survival: basal-like (75%), HER2+(52%), luminal A (84%), and luminal B (87%) [7]. Relatively high Kaplan-Meier survival curves were observed in Luminal A and Luminal B, and relatively steep falls in breast cancer specific survival were observed in the first 4 to 5 years for the basal-like and HER2+ and Basal-like [7].

Our results suggest that the distance or similarity of cancer subtypes based on pathway analysis might be able to filter the noise in gene expression variance or protein intensity change; therefore, focusing on function and interaction between them might provide further insight into the intrinsic relationship of cancer subtypes. Our results also revealed that intrinsic trait combination specific pathway changes may influence tumor progression and be helpful for early detection and early therapeutic intervention.

There are several potential limitations to this study. First, classifications based on ER PR and HER2 status are only approximations of the molecular breast cancer subtypes. Conclusions based on the receptor-based approximations cannot necessarily be applied to the molecular subtypes. For example, Carey et al. [7] reported that this definition for luminal B does not identify all luminal B tumors because only 30% to 50% are HER2 positive, so our approximation of the luminal B group as ER or PR positive and HER2 positive may miscategorize a proportion of the true luminal B group as luminal A. Another possible limitation to the study relates to the relatively small numbers in some subtypes, particularly the luminal B group and HER2+ group, which contained only 15 and 10 patients, respectively, compared to luminal A with 24 patients. However, with the development of our ongoing CPTAC, more patient samples will be collected. And in the future, as the technology of molecular markers improves, more sophisticated markers than ER, PR, and HER2 immunophenotype may become available. Third, there is Unknown subtype instead of normal-like subtype in our data. Although we guess it might possibly be normal-like subtype, it cannot be treated as normal like subtype. Figure 8 shows the improved prediction accuracy after we remove the Unknown subtype. Correctly cataloging those unknown subtypes will help to improve distance measuring and prediction of cancer subtype.

### Breast cancer and other disease pathway

Breast cancer genes, as genes of a complex disease, not only suffer from the disturbance of its local sub-network, but also share some common genetic origin with other related diseases. For example, Gol *et al*. suggested by the cancer sub-network analysis that many cancer phenotypes share common genetic origins with other diseases[33].

Further we investigated the breast cancer-related disease pathways including complement system, pathways in cancer, thyroid cancer, systemic lupus erythematosus, huntington's disease, prion diseases, alzheimer's disease, parkinson's disease, and oxidative phosphorylation (additional file 1).

The complement system consists of a series of about 25 proteins that work to "complement" the work of antibodies in destroying bacteria. The complement system has important protective functions in autoimmune system, but can also, when inappropriately activated, cause tissue damage. Deficiencies in the early pathway components C1, C2 and C4 predispose the development of autoimmune disease such as SLE and SLE-like disorders. In recent years, it has become evident that complement activation is involved in the tumor cytotoxicity[34]. Administration of murine monoclonal antibodies against various tumor antigens in patients leads to antitumor effects including tumor regression and the localizaiton of both C4 and C3 at the tumor site [34]. The ability of murine monoclonal antibodies to mediate antibody-dependent cellular cytotoxicity (ADCC) with human effector cells and cytotoxicity mediated by complement activation makes these antibodyies promising candidates for cancer therapy [35].

In the pathways in cancer, TGFA and ERBB2 are both important regulators of normal mammary gland physiology, and aberrations in their signaling have been associated with breast tumorigenesis [36]. ERBB2 in breast cancer have been approved for clinical use. And the TGFR are potentially amenable to therapies for treatment of human breast disease[37]. In the Huntington's disease, parkingson's disease, oxidative phosphorylation, and alzheimer's disease pathways, CLTC is involved in inflammatory myofibroblastic tumors [38] and lower expression of MAPT is associated with HER2 overexpression [39].

### Permutation test

Most protein identification methods were based on fold change. For example, a fold change or two samples Student's original t test was carried out by comparing physiological changes between normal and disease states to identify serum biomarkers to detect breast cancer [40]. As we know, a fold change method doesn't take the variability of a protein into account and a t-test requires an assumption of normal distribution of data. However, the datasets we used didn't show normal distribution. The log2 transformated intensity values for all 1458 proteins from healthy women were not from a normal distribution (One-sample Kolmogorov-Smirnov test, D = 0.0419, p-value < 2.2e-16). We also found the intensity values from the four breast cancer subtypes and additionally unknown subtype were not from a normal distribution either.

T-test is a parametric test and the permutation process is non-parametric. By using permutation test we made no assumption about the distribution under the null hypothesis. Usually, the assumptions in the null hypothesis are weakened, and it becomes harder to reject.

In addition to validation using a normal quantile plot, if using Student's original definition of the t-test, the two populations being compared should have the same variance. If the sample sizes in the two groups being compared are not equal, Student's original t-test is not robust to the presence of unequal variances[41]. Welch's t-test has been used by most statistics packages such as t.test function in R when the two sample variance is assumed to be different because it is insensitive to equality of the variances regardless of whether the sample sizes are similar. However, if we have no good reason to believe that the population variances are unequal, the result of Student's Original t-test becomes more reliable than that of Welch's t-test. For example, suppose two random samples, (30.02, 29.99, 30.11, 29.97, 30.01, 29.99) and (29.89, 29.93, 29.72, 29.98, 30.02, 29.98) (F test statistics = 0.2122, p-value = 0.1141 > 0.05. We cannot reject the null hypothesis that true difference in variances is equal to 0), the result of Student's original t-test (statistics = 1.959, p-value = 0.078) becomes more suggestive of a difference in the mean for the two populations of samples than that of Welch's t-test(statistics = 1.959, p-value = 0.091).

During the data permutation, even if the two samples could originally have come from the same population, the variance difference between the two permuted samples could change at each resampling. Using only one type of t-test invariably during the permutation process will obviously result in inaccurate statistics of significance. For example, the ttperm function in Category package of R language uses only Welch's t-tests to perform each permutation.

Using the q value change based on the permutation test p value, breast cancer was classified into four breast cancer subtypes and one unknown subtype with HER2+ and basal-like grouped together (Figure 3), whereas with the traditional permutation test, basal-like was classified as Luminal group, same as the dendrogram in the protein change (Figure 2). Our permutation test method is highly robust to the equality of the variances regardless of whether the same sizes are similar and carries more conviction than the other permutation test, which doesn't consider the effect of equality of variances.

## Conclusion

We report for the first time the pathway-assisted clustering of breast cancer plasma samples, using LC-MS/MS proteomics results. Even though proteomics experiment suffer from a general perception of being noisy and highly variable, we show that with proper bioinformatics integration of breast cancer biological context, it is possible to achieve accurate and sensitive breast cancer subtype classifications. We believe pathway analysis performed at the level similar to ours, which include both intra-pathway and inter-pathway analysis, is key to overcoming noises in the data. Our results also show that proteomic pathway-assisted clustering of breast cancer subtypes can provide biological insight into the intrinsic mechanisms and relationships between different breast cancer subtypes. This insight may help researchers develop diagnostic solutions and customized treatment plans, all based on blood sample. We believe integrative pathway-assisted proteomics analysis described here can become a model for reliable classification of other cancer subtypes and can be used for mining information hidden both within a pathway and between pathways for all cancers.

## Competing interests

The authors declare that they have no competing interests.

## Authors' contributions

JYC conceived the initial work and designed the method. FZ developed the distance method and performed the statistical analyses. All authors are involved in the drafting and revisions of the manuscript.

## Supplementary Material

Additional file 1 **pathway protein matrix**. Click here for file

Additional file 2 **comparing between the four distance metrics (protein intensity, q-value, pathway profiling, and distance score function) and pathway profiling with traditional permutation**. Click here for file
